# Assessing probabilistic predictions of ENSO phase and intensity from the North American Multimodel Ensemble

**DOI:** 10.1007/s00382-017-3721-y

**Published:** 2017-05-13

**Authors:** Michael K. Tippett, Meghana Ranganathan, Michelle L’Heureux, Anthony G. Barnston, Timothy DelSole

**Affiliations:** 1 Department of Applied Physics and Applied Mathematics, Columbia University, New York, NY USA; 2 Department of Meteorology, Center of Excellence for Climate Change Research, King Abdulaziz University, Jeddah, Saudi Arabia; 3 Swarthmore College, Swarthmore, PA USA; 4 National Oceanic and Atmospheric Administration/National Weather Service/National Centers for Environmental Prediction, Climate Prediction Center, College Park, MD USA; 5 International Research Institute for Climate and Society, The Earth Institute of Columbia University, Palisades, New York, NY USA; 6 George Mason University, Fairfax, VA USA; 7 Center for Ocean-Land-Atmosphere Studies, Calverton, MD USA

**Keywords:** ENSO, Probabilistic verification, Ensemble forecasting

## Abstract

Here we examine the skill of three, five, and seven-category monthly ENSO probability forecasts (1982–2015) from single and multi-model ensemble integrations of the North American Multimodel Ensemble (NMME) project. Three-category forecasts are typical and provide probabilities for the ENSO phase (El Niño, La Niña or neutral). Additional forecast categories indicate the likelihood of ENSO conditions being weak, moderate or strong. The level of skill observed for differing numbers of forecast categories can help to determine the appropriate degree of forecast precision. However, the dependence of the skill score itself on the number of forecast categories must be taken into account. For reliable forecasts with same quality, the ranked probability skill score (RPSS) is fairly insensitive to the number of categories, while the logarithmic skill score (LSS) is an information measure and increases as categories are added. The ignorance skill score decreases to zero as forecast categories are added, regardless of skill level. For all models, forecast formats and skill scores, the northern spring predictability barrier explains much of the dependence of skill on target month and forecast lead. RPSS values for monthly ENSO forecasts show little dependence on the number of categories. However, the LSS of multimodel ensemble forecasts with five and seven categories show statistically significant advantages over the three-category forecasts for the targets and leads that are least affected by the spring predictability barrier. These findings indicate that current prediction systems are capable of providing more detailed probabilistic forecasts of ENSO phase and amplitude than are typically provided.

## Introduction

The El Niño-Southern Oscillation (ENSO) phenomenon has well-known global climate impacts (Ropelewski and Halpert 1987). The ability to predict the phase of ENSO in advance brings with it the possibility of anticipating societal impacts from weather and climate variability associated with ENSO that include precipitation extremes (Curtis et al. 2007), Atlantic hurricanes activity (Gray 1984), U.S. seasonal climate (L’Heureux et al. 2015) and tornadoes (Allen et al. 2015). ENSO has recently been linked to the risk of civil conflict (Hsiang et al. 2011).

After unsuccessful attempts in the 1970’s (McPhaden et al. 2014), increased understanding and observational data led to the first successful ENSO forecast in 1986 (Cane et al. 1986). By the time of the strong El Niño of 1997–1998, routine real-time ENSO forecasts were being produced by several groups, but the forecasts were issued and evaluated for the most part deterministically, despite the recognition that forecasts were uncertain (Barnston et al. 1999). The International Institute for Climate and Society (IRI; previously the IRI for climate prediction) began issuing quantitative probabilistic ENSO forecasts in March of 2002 (Barnston et al. 2012; Tippett et al. 2012; Barnston and Tippett 2014). The IRI probabilistic forecasts, later in partnership with NOAA’s Climate Prediction Center (CPC), provide probabilities for the occurrence of El Niño, La Niña, or neutral conditions during upcoming 3-month periods. The phase of ENSO is defined using the NINO 3.4 index. Although the definitions of the three categories in the IRI/CPC ENSO forecasts have changed slightly over time, three categories have always been used. Since there is only one forecast category for each ENSO phase, forecasts give the probability of El Niño, La Niña, or neutral conditions but do not contain any explicit ENSO intensity information, for instance, whether a predicted El Niño event will be strong, weak or moderate.

Prediction of ENSO intensity is important because the severity of climate impacts like drought and precipitation extremes can depend on the strength of the ENSO event (Lyon 2004; Lyon and Barnston 2005; Hoell et al. 2016). The prediction of ENSO intensity received new interest with the exceptionally strong El Ninño of 2015–2016 (L’Heureux et al. 2016). Information about the strength of the expected ENSO state can be provided by either forecasting more than three categories or by providing estimates of the complete forecast probability density (Barnston et al. 2015). Here we take the approach of adding ENSO categories. While more detailed ENSO forecasts that include strength information are desirable, a practical issue is whether current prediction systems are capable of such accuracy.

Here we assess the skill of probabilistic ENSO forecasts with three, five and seven categories from the state-of-the-art dynamical coupled models in the North American Multimodel Ensemble (NMME) project (Kirtman et al. 2014). The two probabilistic skill scores used are the ranked probability skill score (RPSS) and the logarithmic skill score (LSS). The calculation of these skill scores for forecasts with varying number of categories is straightforward. However, using these skill scores to compare forecasts with different number of categories raises some interesting issues regarding the interpretation of skill score values. A key issue is how verification measures can be used to determine whether forecasts expressed with more precision (categories) are justified. Clearly, the skill scores of probability forecasts from a grossly uncalibrated forecast ensemble should be expected to decrease as the addition of more categories reveals deficiencies in greater detail. However, the expected behavior of the skill score values as categories are added is less apparent for high-quality ensembles and could depend on the properties of the particular skill score.

RPSS is the weighted average of squared-error skill scores across categories (Bradley and Schwartz 2011), and as such would not depend strongly on the number of forecast categories if the squared-error skill scores vary little with number of categories. In fact, Daan (1985) presented an example in which RPSS varied little as the number of forecast categories was changed. On the other hand, a forecast with many categories provides more detail and that increased level of detail could provide additional value to some users, depending on forecast quality. The LSS, equivalent to relative ignorance (Bröcker and Smith 2007; Smith et al. 2015) and information gain (Peirolo 2011), can be interpreted as a measure of the information content of a forecast relative to a reference forecast (Roulston and Smith 2002), and as an information measure might be expected to be sensitive to the number of forecast categories and level of forecast detail. Moreover, the information content of a forecast might be expected to increase with additional forecast categories. We are aware of no previous study that has quantified the dependence of the LSS on the number of forecast categories. Daan (1985) found that an *information index*, which is equivalent on average to the ignorance skill score (e.g., Siegert et al. 2011; Tödter and Ahrens 2012; Christensen et al. 2015), decreased as the number of forecast categories increased, contrary to the expectation expressed above that information measures should increase with increased detail. Therefore, in addition to assessing the skill of the NMME ENSO forecasts with varying number of categories, we also investigate the behavior of RPSS and LSS with varying numbers of categories using theoretical considerations and an idealized example where forecast reliability and underlying skill can be specified. Our findings help to interpret skill score values from forecasts with differing numbers of forecast categories and are applicable to probabilistic forecasts generally. We also clarify the relation between the LSS and the ignorance skill score, showing that while the LSS increases as reliable forecast categories are added, the ignorance skill score decreases and goes zero in the limit of many forecast categories, regardless of skill level.

The structure of the paper is as follows. Data and methods, including forecast scores, are described in Sect. 2. Some interpretation and properties of the skill scores are given in Sect. 3. The forecast scores are applied to an idealized example in Sect. 4. The probabilistic scores are computed for the NMME in Sect. 5 for three, five, and seven-category forecasts. A summary and conclusions are given in Sect. 6. Detailed calculations are provided in an “Appendix”.

## Data and methods

### Data

We characterize the ENSO state by the NINO 3.4 index, which is the average sea surface temperature (SST) over the Equatorial Pacific region 5$\,^\circ$S–5$\,^\circ$N and 170$\,^\circ$–120$\,^\circ$W (Barnston et al. 1997). Monthly averages of the observed NINO 3.4 index for the period January 1982–August 2016 are computed using data from Reynolds et al. (2002) which are available at http://iridl.ldeo.columbia.edu/expert/SOURCES/.NOAA/.NCEP/.EMC/.CMB/.GLOBAL/.Reyn_SmithOIv2/.monthly/.sst.

Forecast monthly averages of the NINO 3.4 index come from the North American Multimodel Ensemble (NMME) project (Kirtman et al. 2014). The NMME consists of ensemble forecasts from coupled ocean-atmosphere models developed and run by research and operational centers in the U.S. and Canada. Routine real-time NMME forecasts have been produced since August 2011, and there are hindcasts (reforecasts) for each model that include the period 1982–2010. Here we use integrations with start dates from the hindcast period (1982–2010) and the real-time period (2011–2015) and refer to both as forecasts. Models have been retired from the NMME as well as introduced into the NMME over the course of the project. Only models currently in operation are included in the analysis here. Strictly speaking, integrations made during the early part of the real-time period 2011–2015 by models that were added to the NMME later in the lifetime of the project (for instance, the two GFDL FLOR models) are hindcasts, but we do not make that distinction here.

To support real-time forecasting, models are initialized near the start of each month, and forecasts extending up to 12 months into the future are available by the eighth of the month (Kirtman et al. 2014). The initialization method, forecast length and number of ensemble members vary by model. The models used in this study, their number of ensemble members, and the number of forecast leads (in months) are listed in Table 1. CFSv2 has a small gap between the end of its hindcast data and the start of real-time forecast data, and that information is also found in Table 1. We label the monthly averages of a 12-month integration as having lead times of: 0.5, 1.5, ..., 10.5, and 11.5 months so that the 0.5 month lead of a forecast with nominal start date of January 1 is the January average, and so on.

**Table 1:** NMME models, ensemble size, number of forecast leads and data availability

Model	Ensemble size	Forecast length (months)	Available forecasts
GFDL-CM2p1-aer04	10	12	January 1982–December 2015
NASA-GMAO-062012	11^a^	9	January 1982–December 2015
COLA-RSMAS-CCSM4	10	12	January 1982–December 2015
GFDL-CM2p5-FLOR-A06	12	12	January 1982–December 2015
GFDL-CM2p5-FLOR-B01	12	12	January 1982–December 2015
CMC1-CanCM3	10	12	January 1982–December 2015
CMC2-CanCM4	10	12	January 1982–December 2015
NCEP-CFSv2	24^b^	10	January 1982–December 2010, April 2011–December 2015

^a^All months have 11 ensemble members except June when there are 12 ensemble members

^b^Last lead real-time forecasts have 8–12 ensemble members. The DL Jun 2012 start is missing 4 ensemble members (5/31/2012)

The NMME hindcasts are designed to mimic the real-time forecasting process. However, the CFSv2 hindcasts differ from those of other models because they were designed to accommodate the needs of subseasonal (with more frequent forecast issuance) as well as seasonal prediction (Saha et al. 2014). For that reason, the CFSv2 hindcasts starts are provided on every fifth day (pentad) with 4 forecasts per day. For comparison with seasonal predictions issued once per month, these pentad starts are organized as monthly starts that contain the 6 pentad starts prior to the 7th of the month, except for November starts when 7 pentad starts are available. This arrangement means that the CFSv2 hindcasts have at least 24 ensemble members, but that some of those members are initialized in the second week of the month prior to the nominal start date. The situation in real time is different, and four 9-month integrations of the CFSv2 are run daily. For consistency with the hindcast period, we use the same pentad sampling of CFSv2 start dates during the real-time period. Current products from NOAA’s Climate Prediction Center (CPC) do not use the pentad sampling but instead use forecasts starting from the last day of the previous month and the first seven days of the current month, presumably benefiting from more recent initial conditions but potentially with ensemble spread that may differ from that of the hindcasts at short leads.

**Table 2:** Root-mean squared-error (degrees Celsius) of the First-lead NINO 3.4 forecasts

Model	First-lead RMSE
COLA-RSMAS-CCSM3	0.29
GFDL-CM2p1-aer04	0.21
NASA-GMAO-062012	0.21
COLA-RSMAS-CCSM4	0.33
COLA-RSMAS-CCSM4-2c	0.22
GFDL-CM2p5-FLOR-A06	0.22
GFDL-CM2p5-FLOR-B01	0.21
CMC1-CanCM3	0.17
CMC2-CanCM4	0.18
NCEP-CFSv2	0.43
NCEP-CFSv2-2c	0.25
MME	0.17

**Fig. 1:**
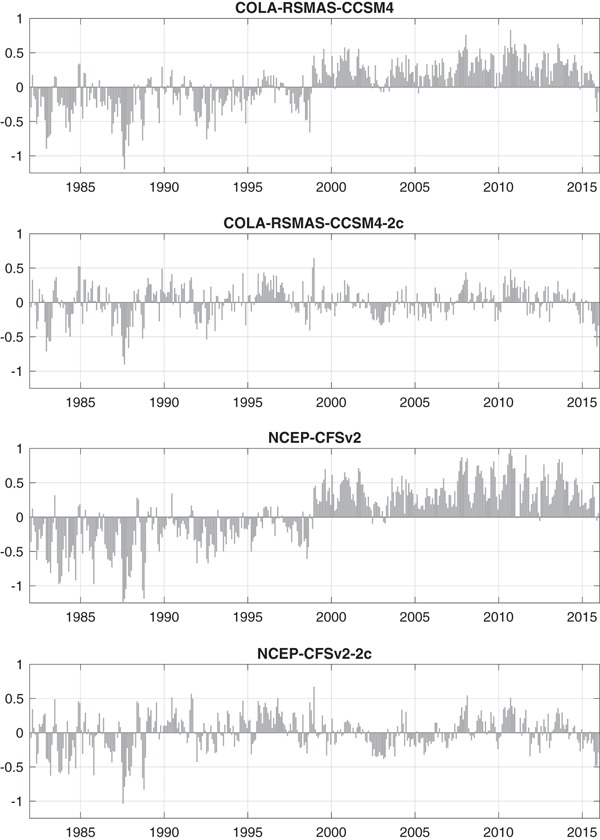
First-lead errors (ensemble mean forecast anomaly−observation anomaly) of 1982–2015. COLA-RSMAS-CCSM4 (1982–2010 climatology), COLA-RSMAS-CCSM4-2c (two climatologies), NCEP-CFSv2 (1982–2010 climatology) and NCEP-CFSv2-2c (two climatologies)

**Fig. 2:**
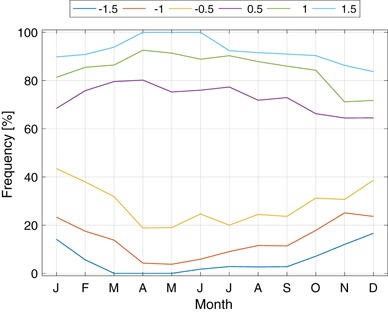
The frequency by calendar month (abscissa) that the NINO 3.4 anomaly ($^\circ$C relative to 1982–2010) is less than −1.5, −1.0, −0.5, 0.5, 1.0, 1.5 for the period 1982–2015

**Fig. 3:**
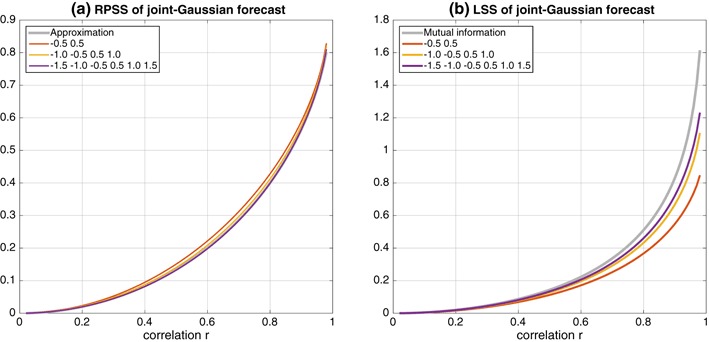
Dependence of **a** RPSS and **b** LSS for joint-Gaussian distributed variables on their correlation *r* for the category boundaries given in the legends. The* curve* in panel **a** labeled “Approximation” is the graph of $1-\sqrt{1-r^2}$ and is nearly hidden by the *curve* for the seven-category forecast. The *curve* in **b** labeled “Mutual information” is the graph of $-\frac{1}{2} \log (1-r^2)$ which is the mutual information of the continuous joint-Gaussian distributed forecasts and observations

**Fig. 4:**
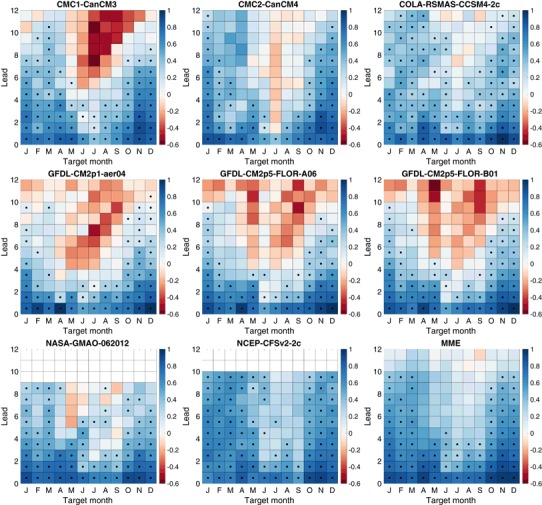
Ranked probability skill score (RPSS) of three-category forecasts. *Black dots* indicate skill that is statistically significantly better (5% significance level) than a climatological forecast

**Fig. 5:**
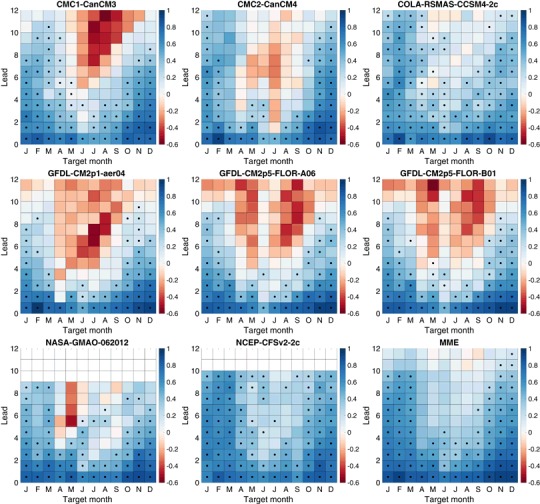
Ranked probability skill score (RPSS) of five-category forecasts. *Black dots* indicate skill that is statistically significantly better (5% significance level) than a climatological forecast

**Fig. 6:**
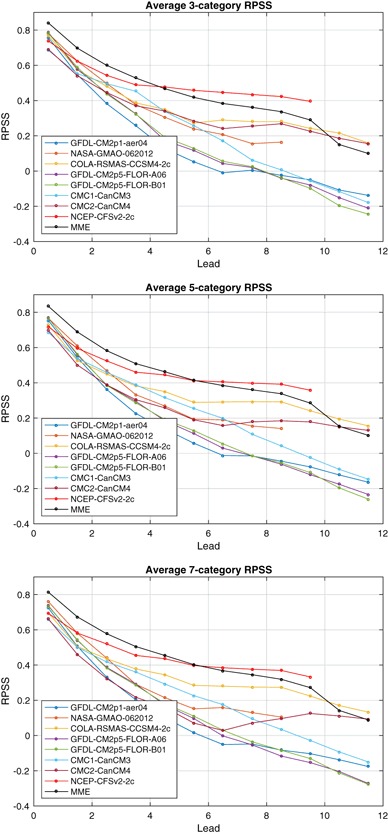
Ranked probability skill score (RPSS) as a function of lead, averaged over all target months for three-category (*top*), five-category (*middle*) and seven-category forecasts (*bottom*). See text for category definitions

**Fig. 7:**
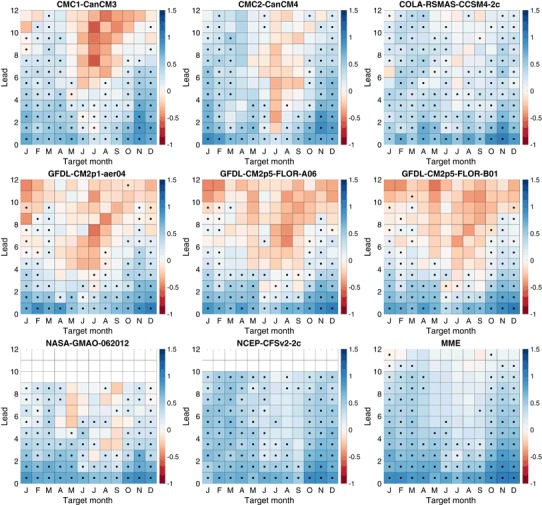
Logarithmic skill score (LSS) of a three-category forecasts. *Black dots* indicate skill that is statistically significantly better (5% significance level) than a climatological forecast

**Fig. 8:**
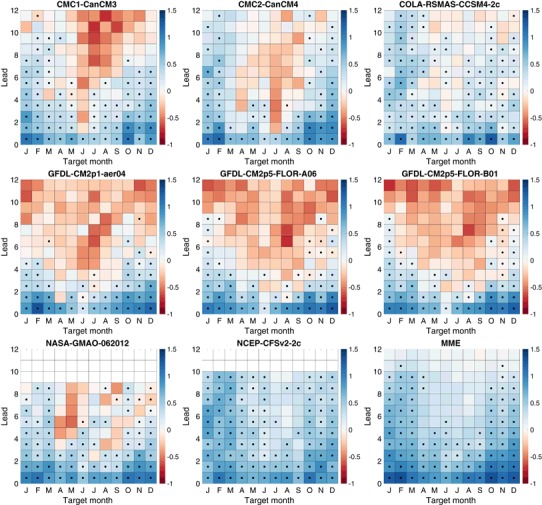
Logarithmic skill score (LSS) of five-category forecasts. *Black dots* indicate skill that is statistically significantly better (5% significance level) than a climatological forecast

**Fig. 9:**
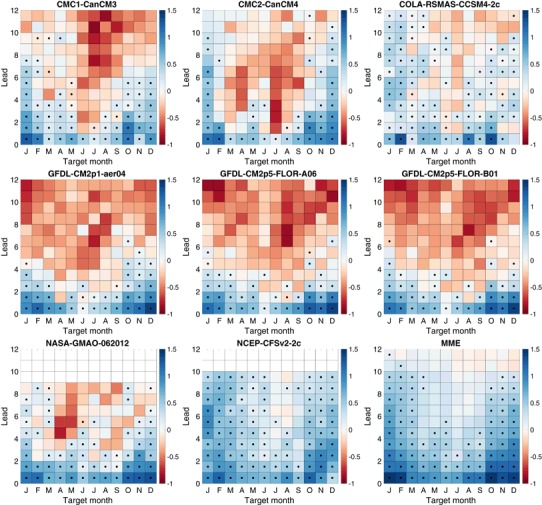
Logarithmic skill score (LSS) of seven-category forecasts. *Black dots* indicate skill that is statistically significantly better (5% significance level) than a climatological forecast

**Fig. 10:**
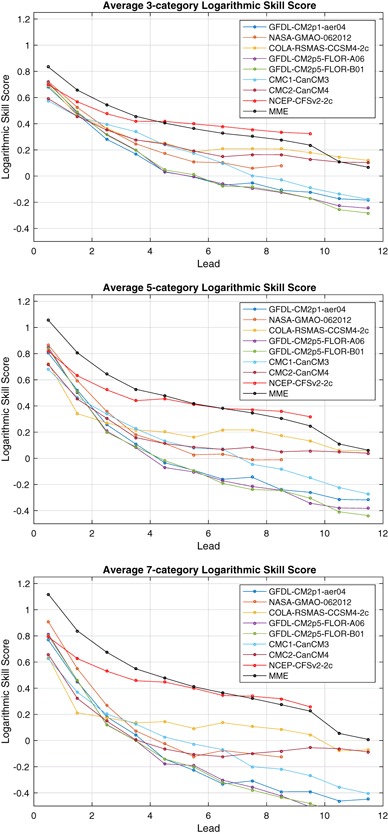
Logarithmic skill score (LSS) as a function of lead, averaged over all target months for three-category (*top*), five-category (*middle*) and seven-category forecasts (*bottom*). See text for category definitions

**Fig. 11:**
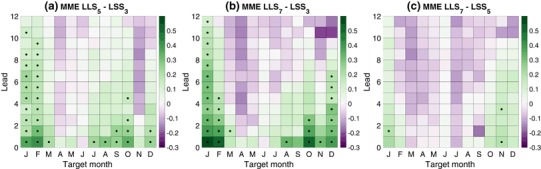
Difference between MME LSS for **a** three and five-category forecasts, **b** three and seven-category forecasts, and **c** five and seven-category forecasts. Positive values indicate increased LSS with more categories. *Black dots* indicate where the increase in LSS is statistically significant at the 5% significance level using a one-sided Wilcoxon signed rank test

**Fig. 12:**
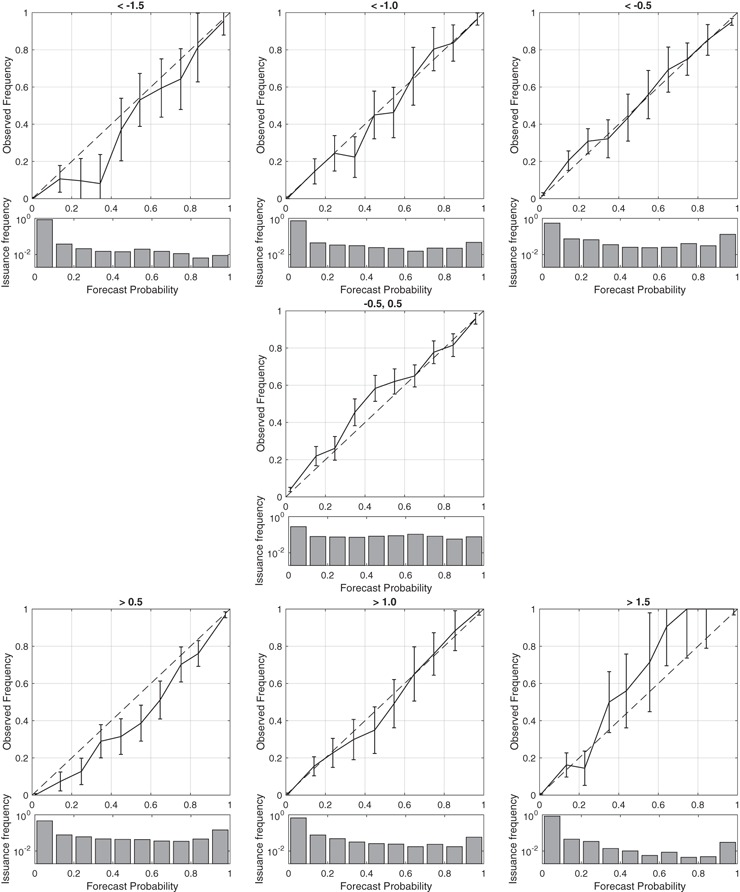
Reliability diagrams and frequency of occurrence histograms for the MME and the categories indicated in the titles. The reliability statistics are computed by pooling the first six forecast leads. *Error bars* are twice the Bernoulli standard deviation for the bin-averaged probability

Monthly anomalies for each model and the observations are computed relative to the 29-year climatology period 1982–2010. By using only anomalies in our analysis, we remove forecast model biases that are stationary with respect to calendar month and forecast lead. However, several studies have noted a discontinuity in the forecast bias of the CFSv2 SST hindcasts around 1999, which is related to the data assimilation and initialization procedure (Xue et al. 2011; Kumar et al. 2012; Barnston and Tippett 2013). The root-mean squared error (RMSE) of the ensemble mean forecast anomalies for the first (0.5 month) forecast lead are given in Table 2 and indicate that the RMSE of the CFSv2 forecasts is roughly twice that of most other models. The time-series of the difference of ensemble mean forecast and observation anomalies (Fig. 1) shows that CFSv2 first-lead forecasts are systematically too cool prior to 1999 and too warm after 1999. CCSM4 has the next highest first-lead RMSE error, and its first-lead errors have a similar bias to that of CFSv2, though to a lesser degree (Fig. 1). This behavior is explained by the fact that CCSM4 shares initial conditions with CFSv2 which come from the climate forecast system reanalysis (Saha et al. 2010; Kirtman et al. 2014; Infanti and Kirtman 2016). One strategy is to compute anomalies for these models using two climatological periods: 1982–1998 and 1999–2010. The two-climatology forecast anomalies, denoted CFSv2-2c and CCSM4-2c, show no apparent non-stationarity in forecast biases (Fig. 1), and we use them in the multimodel ensemble (MME) in place of CFSv2 and CCSM4, respectively. The use of two climatologies does give some advantage to these two models since it guarantees their anomalies have no bias on two periods separately, whereas other model anomalies are only unbiased on a single period (1982–2010). On the other hand, the use of two climatologies means that a shorter climatology period (1999–2010) is used during the real-time period 2011–2015, with a potentially negative impact through greater sampling variability.

CCSM3 also has a large first-lead RMSE (Table 2) but there is no indication of nonstationarity in its first-lead errors (not shown). Kirtman et al. (2014) noted that CCSM3 was somewhat of an outlier in the frequency with which its tropical SST forecast error exceeded that of the multi-model mean. DelSole and Tippett (2016) noted that at lead equal to 2.5 months, CCSM3 had significantly larger squared errors than either a multi-model mean or baseline regression model. Although CCSM3 is currently still being run, NOAA’s Climate Prediction Center has replaced it with CCSM4 in its forecast products, and here we do not include it in the MME.

The MME considered here includes 8 models and has at least 99 ensemble members for the shortest leads, decreasing to 64 at the last lead for most months. The June starts of the NASA model have 12 ensemble members, making a total of 100 members over the first 9 leads in June. We only use 24 CFSv2 members for November starts. The CFSv2 ensemble has fewer than 24 members at its last lead (9.5 months) in the real-time period because the earliest pentad start forecasts do not extend as far into the future as they do in the hindcasts (e.g., the last target of the integrations starting on November 12 in the hindcasts is September of the following year, while the last target in real time integrations is August.).

Hindcast and real-time forecast monthly averages of SST data, as well as near-surface temperature and precipitation are available for download from the IRI Data Library at http://iridl.ldeo.columbia.edu/SOURCES/.Models/.NMME/. Additional variables and higher-frequency (daily) data is available from NCAR’s Earth System Grid https://www.earthsystemgrid.org/search.html?Project=NMME.

### Probability forecasts

In general terms, ENSO probability forecasts give the likelihood that El Niño (warm), neutral or La Niña (cool) conditions will occur in the future. Three-category forecasts for 3-month periods are common. From 2002–2011, IRI issued probability forecasts for ENSO categories defined so that El Niño and La Niña conditions approximately corresponded to the upper quartile and lower quartile, respectively, of the historical distribution of NINO 3.4 values. The advantage of such approach is that NINO 3.4 values are classified consistently throughout the year according to their climatological frequency. Tercile-based category definitions also classify ENSO events based on frequency (Becker and van den Dool 2015). Category definitions based on terciles or quartiles have boundaries in physical units that vary seasonally because the variance of NINO 3.4 is substantially larger in boreal fall/winter than in boreal spring/summer.

On the other hand, a disadvantage of categories defined by percentile thresholds is that the category boundaries expressed in physical units do in fact vary seasonally, are data-dependent, and, for these reasons, are more difficult to communicate to users. The official CPC ENSO definitions and forecasts use fixed category boundaries (${\pm }0.5\,^\circ$C; Kousky and Higgins 2007) which are simpler to explain but whose statistical meaning varies during the year in the sense that a $0.5\,^\circ$C anomaly is a more likely event in December than in April (Fig. 2). The Australian Bureau of Meteorology uses $\pm 0.8\,^\circ$C for its category boundaries (L’Heureux et al. 2016).

CPC sometimes describes the intensity of an ENSO event as “moderate” or “strong,” but does not issue intensity forecasts. Moderate is used informally to mean NINO 3.4 anomalies whose amplitude exceeds $$1.0\,^\circ$$C, and strong to mean those anomalies whose amplitude exceeds $$1.5\,^\circ$$C. Based on this informal practice, we consider here three, five and seven-category ENSO probability forecasts whose category boundaries are, in degrees Celsius with respect to the 1982–2010 climatology, $$[-0.5, 0.5],$$$$[-1.0, -0.5, 0.5, 1.0]$$ and $$[-1.5, -1.0, -0.5, 0.5, 1.0, 1.5]$$, respectively. The width in probability of the middle category varies from about 20% in winter to nearly 60% in spring (Fig. 2).

Forecast ENSO category probabilities are computed from forecast ensembles using

1 $$\begin{aligned} p_i & = \text {Probability} (\,i\text {-th category})\\ &= \frac{\text {Number of ensemble members in the}\,i\text{-th category}\,+\,\frac{1}{C}}{\text {total number of ensemble members}\,+\,1} \end{aligned}$$

where *C* is the number of forecast categories (Tippett and Barnston 2008). Categories probabilities sum to one. The benefit of this approach is that it ensures that forecast probabilities of 100 or 0% do not occur. Ensemble members are weighted equally in the multimodel (MME) which has the effect of giving more weight to models with more members (here CFSv2). There is no attempt to weight models by their skill since unequal weighting given this number of models and sample size tends to be not robust or statistically justified (DelSole et al. 2013).

### The ranked probability and logarithmic skill scores

The ranked probability score (RPS) is used to score probabilistic forecasts of ordered, mutually exclusive events or categories. RPS is a *strictly proper* scoring rule meaning that forecasters maximize their expected score only by forecasting their true beliefs; the score is not open to manipulation or hedging (Wilks 2011). RPS is the sum of the squared differences of the cumulative forecast probabilities and the corresponding outcomes. For a forecast with *C* categories, the cumulative forecast probability $P_i$, expresses the forecaster’s belief that the verifying observation *o* will not exceed the category boundary $c_i$, for $i=1, 2,\dots , C-1$. The cumulative forecast probability $P_i$ is related to the category probability $p_i$ by

2 $$\begin{aligned} P_i = \sum _{j=1}^i p_j\,, \end{aligned}$$

where $p_j$ is the forecast probability of the observation occurring in the *j*-th category. The RPS of a single forecast is

3 $$\begin{aligned} \text {RPS} = \sum _{i=1}^{C-1} (P_i - O_i)^2\,, \end{aligned}$$

where $O_i$ is 1 when $o \le c_i$ and zero otherwise. The ranked probability skill score (RPSS) is defined in terms of the ranked probability score RPS as

4 $$\begin{aligned} \text {RPSS} = 1 - \frac{\overline{\text {RPS}}}{\overline{\text {RPS}}_{\text {ref}}}\,, \end{aligned}$$

where $\overline{\text {RPS}}$ and $\overline{\text {RPS}}_{\text {ref}}$ are the RPS of the forecast being evaluated and a reference forecast, respectively, averaged over multiple forecasts (Eq. 8.52; Wilks 2011). The reference forecast used here has constant cumulative categorical probabilities $Q_i = \overline{O}_i$, for $i=1, 2,\dots , C-1$, where again $\overline{(\cdot )}$ denotes average. For the NMME ENSO forecasts, the averages are computed over the full period 1982–2015. The $Q_i$ are the unconditional probabilities of occurrence of the respective cumulative categories, and for tercile categories, $Q_1=1/3$ and $Q_2=2/3$.

The logarithmic score (LS) is an alternative to the RPS and for an individual forecast is the simply the logarithm of the forecast probability of the observed category (Good 1952; Roulston and Smith 2002). Thus, if the observation falls in the *i*-th category, then the LS of the forecast is $\log p_i$. The LS does not depend on the forecast probabilities of the other categories, and this property means that the LS is a *local* score, unlike RPS, which depends on the probabilities assigned to categories other than the one that occurs. The LS is a strictly proper local scoring rule, and all strictly proper local scoring rules are equivalent to the LS (Gneiting and Raftery 2007). We define the logarithmic skill score (LSS) as

5 $$\begin{aligned} \text {LSS} = \overline{\text {LS}}-\overline{\text {LS}}_{\text {ref}}\,, \end{aligned}$$

where $\overline{\text {LS}}$ and $\overline{\text {LS}}_{\text {ref}}$ are the LS of the forecast being evaluated and a reference forecast, respectively, averaged over multiple forecasts. Here we take the reference forecast to have constant probabilities $q_i = \overline{o}_i$, consistent with the frequency of occurrence reference forecast used to define $\text {RPS}_{\text {ref}}$. Positive values of LSS mean greater skill than the reference forecast, and negative values mean less skill than the reference forecast. Except for the use of natural logarithms, the LSS is the same as the *information gain* (Peirolo 2011) and the negative of the *relative ignorance* (Bröcker and Smith 2007; Smith et al. 2015). The LSS differs from the ignorance skill score that is formed from ratios (rather than differences) of logarithmic scores (e.g., Daan 1985; Siegert et al. 2011; Tödter and Ahrens 2012; Christensen et al. 2015). For a fixed number of categories, the ignorance skill score is proportional to the LSS with

6 $$\begin{aligned} \text {Ignorance skill score} = 1 - \frac{\overline{\text {LS}}}{\overline{\text {LS}}_{\text {ref}}} = - \frac{\text {LSS}}{\overline{\text {LS}}_{\text {ref}}}\,. \end{aligned}$$

However, in contrast to the LSS, the ignorance skill score goes to zero in the limit of many categories, regardless of skill level because $-\overline{\text {LS}}_{\text {ref}}$ is the discrete entropy of the climatological distribution and goes to infinity in the limit of many categories (see discussion in “Appendix A.2”).

## Interpretation of skill score values

In the case of two categories ($C=2$), RPS reduces to the Brier score (Brier 1950). The Brier score is the sum squared error of the probability forecasts, and the Brier skill score (BSS) normalizes that sum squared error relative to a reference forecast. When the reference forecast is the occurrence frequency (as it is here), the BSS is the sum of three quantities (Murphy 1988)

7 $$\begin{aligned} \text {BSS} = \rho ^2 - \left( \rho - \sqrt{\frac{S}{T}} \right) ^2 - \frac{(\overline{p}_1 - \overline{o}_1)^2}{T}\,, \end{aligned}$$

where $\rho$ is the sample correlation between $p_1$ and $o_1$, *S* (“signal”) and *T* (“total”) are the sample variances of $p_1$ and $o_1$, respectively, and $\overline{p}_1$ and $\overline{o}_1$ are the averages of the forecasts and observations. The two negative terms on the right-hand side of (7) are the conditional and unconditional forecast biases and vanish for reliable forecasts. Therefore, the BSS of a reliable forecast is simply the squared correlation between the forecast probabilities and occurrences. This fact is relevant for RPSS, since RPSS is the weighted average of the Brier skill scores of the cumulative probabilities (Bradley and Schwartz 2011). Hence for reliable forecasts, RPSS is a weighted average of squared correlations. As such, we would not expect RPSS to change substantially as categories are divided or combined, as long as the reliability and the degree of association between forecast probability and occurrence is maintained.

One useful interpretation of the LSS comes from imagining a game where forecasters wager on the category in which the observation will fall (Kelly 1956; Cover and Thomas 1991; Roulston and Smith 2002; Hagedorn and Smith 2009). How should forecasters wager given their knowledge of the forecast probabilities? Forecasters can maximize the expected value of their resulting wealth by putting all their money on the category with the highest expected payout. However, this strategy results in zero wealth if the observations falls in another category. Alternatively, forecasters may choose to maximize the expected value of the logarithm of their resulting wealth, choosing the logarithm as a utility function because the logarithm makes zero wealth infinitely unattractive. Suppose the forecasters wager a fraction $f_i$ of their wealth on each of the categories, $i=1,\dots ,C$. If the observation falls in the *i*-th category, their resulting wealth is proportional to $f_i$, and the logarithm of their resulting wealth depends on the quantity $\log f_i$. Now, the problem of choosing the fraction $f_i$ that maximizes the expected logarithm of the wealth of the forecasters is precisely the same as the problem of choosing the probability $p_i$ that maximizes the expected LS of their forecasts. Since the LS is a strictly proper score, the expected value of $\log f_i$ (the logarithm of the wealth of the forecaster) is maximized by taking $f_i=p_i$. This strategy is called “betting your beliefs” because the amount wagered on each category is proportional to the forecaster’s prediction (Poundstone 2010). The wealth of such a gambler increases (or decreases) by the factor $p_i/q_i$ where $q_i$ is the reference probability used to set the odds for the *i*-th category. The logarithm of the wealth of the forecaster depends on the quantity $\log p_i - \log q_i = \text {LS} - \text {LS}_\text {ref}$, which is precisely the LSS of a single forecast. The logarithm of the value of a series of such wagers depends on the difference of the average LS of the forecast and the average LS of the reference forecast, which is the LSS defined in (5).

Since the LSS is a measure of the economic value of a forecast (in the context of the hypothetical wagering game), a comparison of the LSS values of two forecast systems is meaningful, even if the forecast systems have different formats or numbers of forecast categories. The forecast system with the higher LSS is more attractive to gambling forecasters because their winnings are greater.

The LSS also has connections to information theory (Roulston and Smith 2002). The expected LSS of a reliable forecast (see Eq. 16 of the “Appendix”) is the relative entropy between the forecast and the reference probability distributions. Relative entropy is a measure from information theory that quantifies the information difference between two probability distributions and has been used to measure forecast quality and utility (Cover and Thomas 1991; Kleeman 2002; DelSole 2004; Tippett et al. 2004; DelSole and Tippett 2007). Therefore, the expected LSS of a reliable forecast is the information advantage of the forecast probability distribution over the reference distribution. Moreover, relative entropy, and hence the expected LSS of a reliable forecast, always decreases when categories are grouped together (see Eq. 17 of the “Appendix”). For instance, if two adjacent categories are combined, then their forecast probabilities are added, and the expected LSS of the resulting forecast must decrease. This property of the LSS is reasonable since it means that reducing the level of detail in a reliable forecast reduces its expected information advantage.

## RPSS and LSS of an idealized forecast with differing numbers of categories

Before examining the skill scores of ENSO probability forecasts with varying numbers of categories, we examine how such skill scores depend on the number of forecast categories, “all other things being equal.” By all other things being equal, we mean that the forecasts are reliable, category boundaries are nested, and forecast probabilities are consistent. By nested category boundaries, we mean the category boundaries of the forecast with fewer categories are included in the category boundaries of the forecast with more categories. For example, the category boundaries $[-0.5, 0.5]$ are included in $[-1.0, -0.5, 0.5, 1.0]$ but not in $[-1.0, -0.25, 0.25, 1.0]$. With nested categories, the number of forecast categories is increased by dividing existing categories. By consistent, we mean that the forecast probabilities for combined categories are the sum of the forecast probabilities of their constituent categories.

First, we consider a single forecast and compute skill scores for three and five-category forecasts. As dictated by theory, the expected LSS increases as the number of nested categories increases. On the other hand, the expected RPSS actually decreases. Second, we average the skill scores over many forecasts using forecasts and observation that are generated from continuous joint-Gaussian distributed variables. Since the expected LSS of a reliable forecast decreases when categories are combined, the LSS averaged over many forecasts decreases when categories are combined. On the other hand, we observe that RPSS averaged over many forecasts increases slightly when categories are combined.

### Example: a single forecast

Consider a five-category forecast $\mathbf {p} = [0.023, 0.062, 0.37, 0.24, 0.31]$ along with the reference forecast $\mathbf {q} = [0.16, 0.15, 0.38, 0.15, 0.16].$ If the observation falls in the highest category, the RPS of the forecast is 0.69, the RPS of the reference forecast is 1.3 and the RPSS of this forecast is 0.47. Combining the outer two categories leaves a three-category forecast with forecast probabilities $\mathbf {p} = [0.085, 0.37, 0.55],$ and reference forecast probabilities $\mathbf {q} = [0.31, 0.38, 0.31].$ For the same verifying observation in the highest category, the RPS of the three-category forecast is 0.21, the RPS of the three-category reference forecast is 0.57, and the RPSS of the three-category forecast is 0.63. Reducing the number of categories *increases* the RPSS value for this forecast and verifying observation.

On the other hand, for the same forecasts and observation, the LSS of the five-category forecast is 0.67 and the LSS of the three-category forecast is 0.58. The LSS value *decreases* for this forecast and observation when the number of categories is reduced, the opposite behavior as RPSS. Other verifying observations can, and in this example do, result in the LSS increasing when the number of categories is reduced since the theory only requires that the LSS of a reliable forecast decrease on average when categories are combined.

For reliable forecasts, we can use (11), (13) and (16) to compute the expected skill scores. The expected skill scores are the average of the skill scores for the different possible observation outcomes weighted by their likelihoods, which are given by the reliable forecast. Doing so, we find that the expected value of RPSS *increases* from 0.34 to 0.61 as the number of categories is reduced from five to three. Conversely, the expected value of the LSS *decreases* from 0.20 to 0.19 when the number of categories is reduced from five to three.

### Example: Joint-Gaussian forecasts and observations

Now we compute the average skill scores for categorical forecasts and observations that are generated from continuous joint-Gaussian distributed forecast and observation variables. In fact, the categorical probability forecast example of the previous section corresponds to a Gaussian forecast distribution with mean 0.6 and variance 0.64, and a reference forecast (climatology) distribution that is Gaussian with zero mean and unit variance. The five-category boundaries are $\mathbf {c} = [-1.0, -0.5, 0.5, 1.0]$, and the three-category boundaries are $\mathbf {c} = [-0.5, 0.5]$.

When reliable forecast probabilities are generated from joint-Gaussian variables with correlation *r*, the average skill scores depend only on the number of categories and the correlation. (Details of the model and calculations are given in “Appendix A.3”). The average RPSS is shown in Fig. 3a as a function of the correlation *r* for three, five, and seven-category forecasts with boundaries $$[-0.5, 0.5],$$$$[-1.0, -0.5, 0.5, 1.0],$$ and $$[-1.5, -1.0, -0.5, 0.5, 1.0, 1.5],$$ respectively. The dependance of the average RPSS on correlation is well-captured by the approximation (Tippett et al. 2010)

8 $$\begin{aligned} \text {RPSS} \approx 1 - \sqrt{1-r^2}\,. \end{aligned}$$

There is remarkably little dependance of the average RPSS on the number of categories, consistent with the results of Daan (1985). Close inspection shows that the average expected RPSS decreases as the number of categories increases. This behavior means that RPSS values are lower for forecasts with more highly resolved categories, but the same level of underlying skill (correlation *r* of the continuous variables).

The average LSS is shown in Fig. 3b as a function of the correlation *r* for three, five, and seven-category forecasts with the same boundaries as above. The average value of the LSS increases as the number of forecast categories increases. The increase in the LSS is greater at higher correlation levels. The dependance of the expected LSS is bounded by the mutual information (MI) of the continuous forecast and observation variables

9 $$\begin{aligned} \text {MI} = -\frac{1}{2} \log \left( 1 - r^2 \right) \,, \end{aligned}$$

to which it converges in the limit of many categories (see Eq. 18 and discussion of the “Appendix”).

This example illustrates that when using RPSS to compare forecasts with different numbers of forecast categories, we may see decreases in RPSS values as forecast categories are added, but those decreases are not necessarily an indication of reduced forecast quality. On the other hand, although LSS values increase as forecast categories are added, the increase might not be substantial at low skill levels and depends on the forecasts being reliable.

## Skill of NMME ENSO forecasts

We now use RPSS and LSS to assess the skill of categorical probabilistic ENSO forecasts from individual models and the MME. We assess statistical significance for each target and lead using a one-sided sign test with 5% significance level (Hamill 1999; DelSole and Tippett 2014). The sample size is 34 for most targets and leads but is 33 for longer leads that verify in late 2016 and also less than 34 for CFSv2 which has a gap between the hindcast and real-time data. Skill scores are computed for each target calendar month and lead separately to avoid aggregating forecasts with different climatological probabilities (Hamill and Juras 2006).

### Ranked probability skill score of NMME ENSO forecasts

ENSO predictability varies seasonally, and forecast skill depends on the target calendar month as well as the forecast lead. The RPSS of three-category forecasts as a function of target month and lead is shown in Fig. 4 for the eight individual models and the MME. The dominant feature in all of the models and the MME is the so-called northern spring predictability barrier in which forecasts targeting late spring and summer months have little skill at leads more than a couple of months (Jin et al. 2008; Barnston et al. 2012; Larson and Kirtman 2016). The RPSS for the target months of May–September is substantially less than for targets at other times of the year. The RPSS is not statistically significantly greater than zero for these same late-spring through summer target months for forecast leads greater than 4 months, and is negative for many models, indicating average RPS values greater than that of the reference forecast. These negative RPSS values may reflect amplitude biases where forecast signals are disproportionately large relative to their skill level (Barnston et al. 2017). CFSv2 forecasts show statistically significant RPSS values at long leads for May and June targets despite not having statistically significant skill at some shorter leads. This behavior may reflect sampling variability and the large number of statistical significance tests being performed. Or this may reflect some dependence of forecast skill on start month initialization in addition to forecast lead and target, perhaps through differing growth rates of initial conditions (Samelson and Tziperman 2001). In contrast to their behavior for spring and summer targets, many models have positive statistically significant skill for winter target months at leads up to 8 months and up to 10 months in the MME.

The RPSS values of the five-category forecasts shown in Fig. 5 have much the same pattern of skill with respect to target and lead time as those for the three-category forecasts. The RPSS values for targets and leads with significant positive skill show little difference between three and five-category forecasts, though there is some reduction on average for some models. There is a slight tendency for targets and leads with negative RPSS values for three-category forecasts to become slightly more negative for five-category forecasts. Overall there is relatively little difference in the RPSS values of the three and five-category forecasts. A sign test is used to test the null hypothesis that the median of the difference in the RPSS values of the three and five-category forecasts is zero. The null hypothesis can be rejected (two-sided 5% significance level) for only two of the models, CMC2-CanCM4 and CFSv2, which show a statistically significant but modest (0.033 and 0.021, respectively) decrease in RPSS.

The RPSS values of the seven-category forecasts for the individual models and the MME show a similar picture (not shown) with little change in RPSS values as the number of forecast categories is increased. However, the null hypothesis that the median of the difference in RPSS values of the five and seven category forecasts is zero is rejected for all individual models and the MME, with systematically lower RPSS values for seven-category forecasts compared to those for five-category forecasts. This decrease is modest (median of 0.01 for the MME) but consistent across target months and leads.

The three-category RPSS averaged over target months (Fig. 6) is highest for the MME at leads up to 4 months. While benefiting from a larger ensemble size, the MME also benefits from the diversity of predictable signals (DelSole et al. 2014). For longer leads, CFSv2 has an advantage. The MME advantage extends up to 5 months in the five and seven-category forecast. The five-category and seven-category RPSS values averaged over target months are very similar to three-category values (Fig. 6), but with some modest decreases in RPSS as the number of categories increases.

Overall, the behavior of the RPSS-based assessments of NMME ENSO predictions with varying numbers of categories is similar to that of the joint-Gaussian example of Sect. 4.2. The NMME ENSO RPSS values show little sensitivity to the number of categories, and RPSS values show a slight but systematic decrease as the number of categories is increased from five to seven. The joint-Gaussian example of Sect. 4.2 suggests that the systematic decrease in RPSS values of NMME ENSO forecasts as the number of categories is increased from five to seven could be due in part to the dependence of RPSS on the number of categories rather than due to decreased quality. While the example only treated skillful reliable forecasts, NMME ENSO forecasts are not as skillful and reliable for targets and leads most affected by the spring predictability barrier. We see no substantial change in RPSS values as the number of categories varies for these forecast target and leads either. This behavior would indicate that the degree to which RPSS penalizes forecasts lacking skill and reliability does not depend strongly on the number of forecast categories.

### Logarithmic skill score of NMME ENSO forecasts

The three-category LSS values (Fig. 7) show much the same pattern of skill as a function of lead and target as RPSS, with the dominant feature being the clear signature of the spring predictability barrier. LSS values are judged to be statistically significant at leads that are about a month longer than those for RPSS. However, there are substantial differences in the LSS values of the three and five-category forecasts (Fig. 8). Some positive values become more positive, especially at short leads, and most negative values become more negative, most noticeably for the target and leads affected by the spring predictability barrier. This pattern of change with increasing number of categories is also seen in the LSS of the seven-category forecasts (Fig. 9). Our interpretation of this behavior is that the LSS rewards the use of more forecasts categories for targets and leads that are skillfully predicted, while penalizing the use of additional forecast categories for targets and leads in which forecasts lack skill or reliability, and the penalty imposed by the LSS is harsher than that of RPSS. The models least affected are CCSM4-2c, CFSv2-2c and the MME.

The LSS averaged over all targets for three, five and seven-category forecasts shows that the advantage of the MME and CFSv2-2c over other models, and to a lesser extent that of CCSM4-2c, grows as the number of forecast categories increases (Fig. 10). This finding suggests that these models are sufficiently skillful and have enough ensemble members to make more precise forecasts and that the LSS rewards that precision with increased values. Models and leads with poor skill, even if significant and positive on average, have decreased LSS when additional categories are added, meaning that the addition of more categories actually reduces the advantage of the forecast with respect to the reference forecast, in contrast to the behavior of RPSS which changes little. At shorter leads, most of the individual models, unlike the MME, show little gain or loss from adding more categories, perhaps due to their fairly small ensemble size.

Focusing on the MME forecasts, Fig. 11 shows the change in the LSS of the MME as a function of target month and lead as categories are added. The LSS increases as categories are added for the targets and leads in which skill is highest and again reflects the effects of the northern spring predictability barrier. Winter target months (December–February) show the largest increases in the LSS as the number of categories increases from three to five and from three to seven, with the differences being statistically significant even at some long leads (greater than 6 months). There are increases of the LSS in going from three to five categories and from three to seven categories in late summer and fall (July–November), but those increases are statistically significant only at some of the shorter leads. Increasing the number of categories results in no statistically significant increases of the LSS for the target months of April–June.

Our theoretical findings indicate that the LSS should increase as long as there is underlying skill and as long as forecasts are reliable. To assess the reliability of MME forecasts, we compute $E[O_i|P_i]$ (reliability diagrams) for the first 6 leads of MME forecasts for the full set of seven-category boundaries (Fig. 12). The overall reliability is good with the greatest deviation between forecast probabilities and occurrence frequencies present in the most extreme positive category (NINO 3.4 anomaly values greater than 1.5$\,^\circ$) where there is under-confidence (the occurrence frequency exceeds the forecast probability) and considerable sampling variability due to the relatively small number of events.

## Summary and conclusions

Over the last decade and a half, the issuance of probabilistic ENSO forecasts has become routine. Typically, these forecasts take the form of probabilities for the occurrence of El Niño, La Niña and neutral conditions. IRI/CPC forecasts and monitoring products currently use anomalies of the NINO 3.4 index to characterize the ENSO state with values greater than 0.5 $^\circ$C necessary for El Niño conditions, below $-0.5\,^\circ$C for La Niña conditions and intermediate values being considered neutral. Forecasts for the likelihood of these three categories do not contain explicit information about the expected ENSO amplitude, although there may well be a relation between forecast certainty and intensity (Kumar et al. 2000; Tippett et al. 2007). Several studies have argued that climate responses to ENSO are sensitive to the strength of El Niño events (Lyon 2004; Lyon and Barnston 2005; Hoell et al. 2016). Consequently ENSO forecasts with information about intensity are desirable. The question is then whether current forecast models are capable of greater precision.

More detailed ENSO forecasts might take the form of categorical forecasts with more than three categories or might be in the form of continuous probability density functions (Barnston et al. 2015). Here we have examined the probabilistic skill of monthly ENSO forecasts with three, five, and seven categories constructed from North American Multimodel Ensemble (NMME) integrations. The additional categories come from adding thresholds at $\pm 1.0$ and $\pm 1.5\,^\circ$C, corresponding to the thresholds used informally to define moderate and strong events. We used the ranked probability skill score (RPSS) and the logarithmic skill score (LSS) to measure forecast quality. The LSS is equivalent to relative ignorance (Bröcker and Smith 2007; Smith et al. 2015) or information gain (Peirolo 2011). On the face of it, computing and comparing these scores for forecasts with varying numbers of categories is straightforward. However, using these scores to compare forecasts with different formats raises the question of how to interpret skill score values for forecasts with differing numbers of forecast categories. For instance, are skill scores values expected to increase, decrease or remain the same as the number of forecast categories increases? Here we investigated the question of how skill score values depend on the number of forecast categories using theoretical considerations, an idealized example and the NMME ENSO forecasts.

Theory shows that RPSS is the weighted average of squared error skill scores across cumulative probabilities (Bradley and Schwartz 2011) and as such would be expected to be relatively insensitive to the number of forecast categories, to the extent that the skill of predicting the cumulative probabilities does not change much as additional categories are added. These squared error skill scores are equal to the squared correlation between forecast and observations for reliable forecasts, and again it seems reasonable to expect that RPSS would vary little as forecast categories were added, all other factors being equal. The expectation that RPSS is insensitive to the number of forecast categories is confirmed in an example in which the forecasts and observations are generated from a continuous bivariate Gaussian distribution with specified correlation. For fixed correlation, RPSS values change little for forecasts with three, five and seven categories with RPSS slightly declining as the number of categories increased.

We were able to prove that the LSS increases when reliably forecast categories are added. This finding is consistent with our intuition that forecast information increases if its level of detail increases without loss of reliability and with the interpretation of the LSS as either a measure of information or as the economic value of a series of wagers (Roulston and Smith 2002; Hagedorn and Smith 2009). For reliable forecasts, the LSS is the relative entropy between the forecast and climatological distributions, and thus measures how different the forecast distribution is from the climatological distribution. The LSS averaged over reliable forecasts is equal to the mutual information and in the limit of many categories converges to the mutual information of the underlying continuous distributions. These theory-based findings were confirmed in the idealized example which also showed that the convergence of the LSS to the continuous mutual information was slowest when skill was high, meaning that the advantage of many categories is greatest when skill is high. The ignorance skill score, a verification measure related to the LSS (e.g., Siegert et al. 2011; Tödter and Ahrens 2012; Christensen et al. 2015), decreases to zero as forecast categories are added, regardless of skill level. The reason for this behavior is that the discrete entropy of the climatological distribution is used as a normalizing factor in the ignorance skill score, and this quantity goes to infinity in the limit of many forecast categories.

This understanding of the differing sensitivity of RPSS and LSS to forecast format helps to interpret our results for NMME ENSO forecasts with varying numbers of forecast categories. The main feature present in our assessment of the NMME ENSO probability forecasts is the impact of northern spring predictability barrier, which limits how far in advance ENSO conditions in spring and summer can be predicted. Overall, the MME shows the highest level of skill according to all scores with the CFSv2 forecasts also standing out for their skill, after accounting for a discontinuity in their initial conditions and climatology. The two skill scores used, RPSS and LSS, agree on many features such as relative model skill and the seasonality of skill. Monthly NMME ENSO forecasts with three, five, and seven categories showed comparable overall accuracy as measured by RPSS, which can be interpreted as indicating no overall loss of skill with increasing numbers of categories. The examination of LSS indicates that, when supported by a sufficient skill level (which varies by model, target and lead), adding forecast categories can increase LSS values. In particular, the five and seven-category multimodel ensemble (MME) forecast showed increased LSS for the targets and leads least affected by the spring predictability barrier. Conversely, additional forecast categories for targets and leads with low or no skill were harshly penalized by LSS, more so than by RPSS. These findings provide evidence supporting the feasibility of ENSO forecast products with more than three categories.

## Appendix: The average RPSS and LSS of joint-Gaussian distributed forecasts and observations

### A.1: The expected value of RPS

For a single forecast, the expected value (conditional on the forecast) of the RPS is the sum of each possible outcome of (3) weighted by its conditional likelihood:

10 $$\begin{aligned} E[\text {RPS} | P_1,P_2,\dots ,P_{C-1} ] = \sum _{i=1}^{C-1} E[O_i | P_i] (P_i - 1)^2 + (1-E[O_i | P_i]) P_i ^2\,. \end{aligned}$$

If the forecast probabilities are reliable, then $E[O_i|P_i]=P_i$, which means that the reliability diagrams for each category are straight lines with unit slope and zero intercept. The expected RPS of a reliable forecast is

11 $$\begin{aligned} E[\text {RPS} | P_1,P_2,\dots ,P_{C-1} ] = \sum _{i=1}^{C-1} P_i (P_i - 1)^2 + (1-P_i) P_i^2 = \sum _{i=1}^{C-1} P_i (1 -P_i)\,, \end{aligned}$$

which is the sum of the variances of $C-1$ Bernoulli-distributed variables with success probability $P_i$.

The climatological forecast is reliable, and using (11), its expected RPS is

12 $$\begin{aligned} E[\text {RPS}_{\text {ref}}] = \sum _{i=1}^{C-1} Q_i (1 - Q_i)\,, \end{aligned}$$

where $Q_i$ are the probabilities of occurrence of the cumulative categories. The expectation in (12) is an unconditional one because the climatological forecast is constant. The expected RPS of the climatological forecast is 4/9 in the familiar case of tercile categories with climatological frequencies of 1/3. The expected RPS of the reference forecast conditional on a reliable forecast $P_1,P_2,\dots ,P_{C-1}$ is, using (10),

13 $$\begin{aligned} \begin{array}{ll} E[\text {RPS}_{\text {ref}} | P_1,P_2,\dots ,P_{C-1} ] &{} = \displaystyle \sum _{i=1}^{C-1} P_i (Q_i - 1)^2 + (1-P_i) Q_i^2 \\ &{} = E[\text {RPS} | P_1,P_2,\dots ,P_{C-1} ] + \displaystyle \sum _{i=1}^{C-1} (P_i-Q_i)^2 \,. \end{array} \end{aligned}$$

Because the second term on the right-hand side of (13) is nonnegative, the expected RPS of a reliable forecast is always less than the expected RPS of the climatological forecast.

### A.2: The expected value of the LSS

The logarithmic skill score (LSS) of a single forecast with *C* categories is

14 $$\begin{aligned} \text {LSS} = \sum _{i=1}^C o_i \log \frac{p_i}{q_i}\,, \end{aligned}$$

where $p_i$ and $q_i$ are the forecast and climatological probabilities of the *i*-th category, respectively, and $o_i$ is one when the observation falls in the *i*-th category and zero otherwise. We use the convention that $0 \log 0 = 0$. The expected LSS of a single forecast is

15 $$\begin{aligned} E[\text {LSS} | p_1,p_2,\dots ,p_C] = \sum _{i=1}^C E [o_i|p_i] \log \frac{p_i}{q_i}\,, \end{aligned}$$

and the expected LSS of a reliable forecast is

16 $$\begin{aligned} E[\text {LSS} | p_1,p_2,\dots ,p_C] = \sum _{i=1}^C p_i \log \frac{p_i}{q_i}\,, \end{aligned}$$

since for a reliable forecast, $E [o_i|p_i]=p_i$. The right-hand side of Eq. 16 is the Kullback-Leibler divergence (relative entropy) from the climatological distribution to the forecast distribution, which measures the information advantage of the forecast over the climatological distribution.

The expected LSS of a reliable forecast decreases when categories are combined. For instance, suppose the first two categories are combined. The expected LSS of the forecast with the combined category is always smaller than that of the original forecast because

17 $$\begin{aligned} (p_1 + p_2) \log \frac{p_1+p_2}{q_1+q_2} \le p_1 \log \frac{p_1}{q_1} + p_2 \log \frac{p_2}{q_2} \,, \end{aligned}$$

with equality if and only if $p_1/q_1 =p_2/q_2$ (log sum inequality, e.g., 16.3 of Cover and Thomas 1991). Conversely, the expected LSS of a reliable forecast increases when categories are divided and the number of categories increases.

Averaging Eq. 16 over reliable forecasts gives

18 $$\begin{aligned} E[\text {LSS} ] = E\left[ \sum _{i=1}^C p_i \log \frac{p_i}{q_i} \right] \,, \end{aligned}$$

where the right-hand side of (18) is the *mutual information* (DelSole 2004). If the discrete distributions are quantized continuous distributions, then the expected value of the LSS of reliable forecasts converges to the mutual information of the continuous distributions in the limit of many categories (see Section 8.5 of Cover and Thomas 1991).

The information index (INFO; Daan 1985) for a single forecast is defined as

19 $$\begin{aligned} \text {INFO} = 1 - \frac{\sum _{i=1}^C o_i \log p_i }{\sum _{i=1}^C q_i \log q_i }\,, \end{aligned}$$

and can be written in terms of the LSS as

20 $$\begin{aligned} \text {INFO} = 1 - \frac{\text {LSS} + \sum _{i=1}^C o_i \log q_i }{\sum _{i=1}^C q_i \log q_i } = \frac{\text {LSS} + \sum _{i=1}^C (o_i-q_i) \log q_i }{-\sum _{i=1}^C q_i \log q_i } \,. \end{aligned}$$

Taking the expectation gives

21 $$\begin{aligned} E[\text {INFO}] = \frac{E[\text {LSS}]}{\displaystyle -\sum _{i=1}^C q_i \log q_i } \,, \end{aligned}$$

since $E[o_i] = q_i$; the same relation holds for expectations conditional on the forecast. The numerator on the right-hand side of (21) is the discrete entropy of the climatological distribution, which increases without bound as the number of categories increases (e.g., Theorem 8.3.1 of Cover and Thomas 1991). Therefore, INFO is zero in the limit of many categories, regardless of skill level. The relation between INFO and LSS in (21) supports the finding of Daan (1985) found that INFO decreases as the number of categories increases. The expected value of the ignorance skill score as defined by Siegert et al. (2011), Tödter and Ahrens (2012), Christensen et al. (2015) has the same expected value as the information index and also decreases as the number of categories increases, and goes to zero in the limit of many categories, regardless of skill.

### A.3: The average RPSS and LSS for joint-Gaussian distributed variables

Consider forecasts and observation that are joint-Gaussian distributed with correlation *r*. Specifically, suppose that the observation *o* is given by

22 $$\begin{aligned} o = \mu + \epsilon \end{aligned}$$

where $\mu$ is the forecast mean (“signal”) with distribution $\mu \sim N(0,r^2)$, $\epsilon$ is a random noise with $\epsilon \sim N(0,\sigma ^2)$. The noise variance $\sigma ^2$ is related to the correlation *r* of the forecast mean and observation by $\sigma ^2 = 1 -r^2$ with $r^2 \le 1$. In this model, the unconditional (climatological) distribution of the observation *o* is a Gaussian with zero mean and unit variance.

The $C-1$ forecast cumulative categorical probabilities $P_i$ are

23 $$\begin{aligned} P_i = \text {Prob} \{ o \le c_i \} = \Phi \left( \frac{c_i - \mu }{\sigma } \right) \,, \end{aligned}$$

where $c_i$ are the $C-1$ boundaries of the *C* categories and $\Phi$ is the cumulative distribution function of a Gaussian distribution with zero mean and unit variance.

The average value of the RPS for reliable forecasts is computed by averaging (11) over forecasts or equivalently over values of the mean forecast $\mu$,

24 $$\begin{aligned} E[\text {RPS} ] & = \int _{-\infty }^{\infty } \left( \sum _{i=1}^{C-1} P_i (1 -P_i) \right) p(\mu ) \; d\mu \\ &= \int _{-\infty }^{\infty } \left[ \sum _{i=1}^{C-1} \Phi \left( \frac{c_i - \mu }{\sigma } \right) \left( 1 - \Phi \left( \frac{c_i - \mu }{\sigma } \right) \right) \right] p(\mu ) \; d\mu \,, \end{aligned}$$

where the Gaussian probability distribution function for $\mu$ is

25 $$\begin{aligned} p(\mu ) = \frac{1}{r \sqrt{2\pi }} e^{-\mu ^2/2r^2}\,. \end{aligned}$$

The integral in (24) provides the functional dependence of the average RPS on the correlation *r* as well as the category thresholds $c_i$, but it not easy to integrate in closed form.

A Monte Carlo approach to the evaluation of (24) is to simulate *o* and $\mu$ many times using (22) and average the resulting RPS values. The Monte Carlo approach is simple and direct but inefficient and requires a large number of samples. Another approach is to make the change of variable $y = \Phi (\mu /r)$ so that the bounds of the integral become 0 and 1, noting that $dy = p(\mu ) d\mu$,

26 $$\begin{aligned} E[\text {RPS} ] = \int _{0}^{1} \left[ \left[ \sum _{i=1}^{C-1} \Phi \left( \frac{c_i - r\Phi ^{-1}(y)}{\sigma } \right) \left( 1 - \Phi \left( \frac{c_i - r\Phi ^{-1}(y)}{\sigma } \right) \right) \right] \right] \; dy \,, \end{aligned}$$

and a numerical integration method such as the midpoint rule can be used. This approach is more efficient than the Monte Carlo method and is equivalent to sampling equally-spaced points in probability units. A standard numerical method for numerically evaluating the integral in (24) is Gauss–Hermite quadrature, which for any function $G(\mu )$ is the approximation

27 $$\begin{aligned} \int _{-\infty }^{\infty } G(\mu ) p(\mu ) \; d\mu = \frac{1}{r \sqrt{2\pi }} \int _{-\infty }^{\infty } G(\mu ) e^{-\mu ^2/r} d\mu \approx \frac{1}{\sqrt{\pi }} \sum _{i=1}^N w_i G(r \sqrt{2} x_i )\,, \end{aligned}$$

where $x_i$ and $w_i$ are the Gauss–Hermite nodes and weights, respectively, for order *N*. Here we show results computed with $N=48$.

The average of the expected value of the LSS for reliable forecasts is computed by averaging (16) over all forecasts or equivalently over the values of $\mu$

28 $$\begin{aligned} E[\text {LSS} ] = \int _{-\infty }^{\infty } \left( \sum _{i=1}^C p_i \log \frac{p_i}{q_i} \right) p(\mu ) \; d\mu \,. \end{aligned}$$

Like the average of the expected RPS, this improper integral can be estimated by Monte Carlo simulation, numerical integration of a proper integral on a finite interval or Hermite–Gauss quadrature. We use Hermite–Gauss quadrature with $N=48$.
